# What Has 30 Years of HIV Vaccine Research Taught Us?

**DOI:** 10.3390/vaccines1040513

**Published:** 2013-10-30

**Authors:** José Esparza

**Affiliations:** Bill & Melinda Gates Foundation, PO Box 23350, Seattle, WA 98102, USA; E-Mail: Jose.Esparza@gatesfoundation.org; Tel.: +1-206-709-3659; Fax: +1-206-709-3170

**Keywords:** HIV vaccines, lessons learned, clinical trials, HIV, AIDS

## Abstract

When HIV was discovered and established as the cause of AIDS in 1983–1984, many people believed that a vaccine would be rapidly developed. However, 30 years have passed and we are still struggling to develop an elusive vaccine. In trying to achieve that goal, different scientific paradigms have been explored. Although major progress has been made in understanding the scientific basis for HIV vaccine development, efficacy trials have been critical in moving the field forward. Major lessons learned are: the development of an HIV vaccine is an extremely difficult challenge; the temptation of just following the fashion should be avoided; clinical trials are critical, especially large-scale efficacy trials; HIV vaccine research will require long-term commitment; and sustainable collaborations are needed to accelerate the development of an HIV vaccine. Concrete actions must be implemented with the sense of urgency imposed by the severity of the AIDS epidemic.

## 1. Introduction

The development of an HIV vaccine has been a long and tortuous process that, thus far, has consumed nearly 30 years of intense laboratory and clinical work. When HIV was discovered and established as the cause of AIDS in 1983–1984 [1], many people believed that a vaccine would be easily developed and rapidly deployed. After all, vaccinologists had been very successful in developing vaccines for a whole range of viral diseases. 

However, the paradigm that allowed the development of most existing viral vaccines, which is based on the recreation of the protective immunity that develops after natural infection, does not work in the case of HIV. In AIDS, virus-induced immune responses are not capable of preventing re-infection or are very inefficient in slowing progression to disease. 

The history of HIV vaccine development has been a *tour de force* in trying to develop protective immune responses that nature has not learned to produce. Although we are closer today to an HIV vaccine than we were in 1983, it is not possible to predict when we will have a vaccine with sufficient efficacy for use in public health programs. Nevertheless, if we learn lessons from the past and, most importantly, have the wisdom to apply them, we may be able to accelerate the development of a much needed HIV preventive vaccine.

This article summarizes past efforts made to develop a preventive HIV vaccine. It discusses the insights that have guided those efforts, and identifies lessons that can inform the path forward.

This discussion is based on personal experiences after more than 25 years of involvement in the global HIV vaccine effort. First at the World Health Organization (WHO), and the Joint United Nations Program on AIDS (UNAIDS) in Geneva, Switzerland (from 1986 to 2004), and more recently at the Bill & Melinda Gates Foundation in Seattle, WA, United States (since 2004). 

## 2. A Very Brief History of the Global Effort to Develop an HIV Vaccine

I recently reviewed the history of HIV vaccine development [2], and this section presents a summary. Table 1 includes some of the key events in the basic science, clinical trials and organizational fronts.

**Table 1:** Key events on the history of HIV vaccine research and development.

Year	Scientific Progress	Clinical trials	Organizational aspects
1983–1985	HIV identified as the cause of AIDS; rapid advances in the molecular biology of HIV; experimental HIV infection of chimpanzees; first SIV isolated; neutralizing antibodies described.		US Secretary of Health “predicts” clinical trials of HIV vaccines in two years.
1986	Laboratory development of vaccinia vectors for HIV.	First HIV vaccine trial in the world (by Daniel Zagury, in Zaire).	Starting of the neutralizing antibody paradigm wave; NIH establishes the Division of AIDS (DAIDS); First “Cent Gardes” meeting in France.
1987	HIV Envelope glycoproteins identified as targets of neutralizing antibodies.		Launching of the UK Directed AIDS Research Programme.
1988	Initial description of R5 and X4 virus phenotypes.	First HIV vaccine trial in the US (recombinant gp160, MicroGeneSys); Second HIV vaccine trial in the US (gp160 in a vaccinia vector, BMS/Oncogen).	NIH establishes the Office of AIDS Research (OAR); launching of NIH AIDS Vaccine Evaluation Group (AVEG).
1989	Monkey protection experiments with whole inactivated SIV (later found to be mediated by host antigens).		
1990	V3 loop “identified” as Principal Neutralization Domain (PND); Chimpanzee protection with envelope vaccines; development of SHIV.		WHO establishes HIV Vaccine Advisory Committee (selection of international vaccine evaluation sites in Brazil, Rwanda, Thailand and Uganda).
1991	Identification and classification of HIV clades (subtypes).		
1992	Development of live attenuated (nef-deleted) SIV vaccines; DNA immunization reported; isolation of first broadly neutralizing monoclonal antibody (bnmAb).		French ANRS vaccine program, focusing on ALVAC (canarypox) candidate vaccines; WHO establishes Network for HIV Isolation and Characterization; First National Plan for HIV Vaccine Development in Thailand
1993		First clinical trial with an ALVAC vector; clinical trials of V3 peptide vaccine in the US and China (UBI).	NIH “HIV Network for Prevention Trials” (HIVNET); NIH “Preparation for AIDS Vaccine Evaluation” (PAVE); review of NIH HIV vaccine activities.
1994	Initial suggestion that HIV clades may define immunotypes; finding that antibodies induced by vaccines do not neutralize primary isolates.	First trial in Thailand with a V3 peptide vaccine (UBI).	
1995	R5/X4 phenotype explained by HIV binding to second receptor (CXCR4/CCR5).	First trial in Thailand of a gp120 vaccine.	Establishment of the AIDS Vaccine Advocacy Coalition (AVAC).
1996			Publication of the NIH Levine Report; establishment of the NIH AIDS Vaccine Research Committee (the Baltimore Committee); launching of the International AIDS Vaccine Initiative (IAVI).
1997			President Clinton challenge to develop an HIV vaccine in 10 years.
1998	First crystal structure of gp120.	Initiation of phase III vaccine trial in the US (gp120 BB, VAX004, VaxGen).	
1999	Live-attenuated SIV found to cause disease in newborn monkeys.	Initiation of phase III vaccine trial in Thailand (gp120BE, VAX003, VaxGen); initiation of phase I clinical trials in Uganda with an ALVAC vector.	Establishment of the US HIV Vaccine Trials Network (HVTN); launching of the NIH Vaccine Research Center (VRC); establishment of the South African AIDS Vaccine Initiative (SAAVI).
2000			Conception of the African AIDS Vaccine Programme (AAVP); first AIDS Vaccine Conference (Paris)
2003		Negative results of phase III VaxGen gp120 trials; initiation of RV144 trial in Thailand.	End of the antibody paradigm and beginning of the CMI/CTL paradigm; proposal to establish the Global HIV Vaccine Enterprise.
2004	Introduction of repeat low-dose mucosal challenge in NHP.	Initiation of STEP trial in the US (Ad 5 vector).	
2005			Launching of the NIH-funded Center for HIV Vaccine Immunology (CHAVI).
2006			Launching of the Gates-supported Collaboration for AIDS Vaccine Discovery (CAVD).
2007		Initiation of Phambili trial in South Africa (Ad5 vector); stopping of the STEP and Phambili trial for lack of efficacy.	End of the CTL paradigm and beginning of the current wave aimed at inducing more complex immune responses.
2009	A plethora of novel broadly neutralizing antibodies (bnmAb) began to be reported.	RV144 trial reveals 31.2% efficacy; initiation of HVTN 505 (DNA + Ad 5 vector).	
2011	Description of early control of SIV using a Rhesus cytomegalovirus (RhCMV) vectored vaccine that elicits effector memory cells.		Launching of the AIDS Vaccine for Asia Network (AVAN).
2012	Description of subunit organization of membrane-bound HIV trimer; new insights on the mechanism for development of broadly neutralizing antibodies; report of immune correlates in RV144.		
2013		Stopping of HVTN 505 for lack of efficacy.	13th and last AIDS Vaccine Conference (Barcelona)

### 2.1. First Wave of HIV Vaccine Trials: Induction of Neutralizing Antibodies (1986–2003)

The initial efforts to develop an HIV vaccine were based on the concept that neutralizing antibodies would be sufficient to protect against HIV infection. These efforts followed the paradigm established by the licensure in 1986 of the first recombinant vaccine, against hepatitis B. After all, most existing vaccines work through antibodies that block infection or interfere with systemic infections [3]. Different HIV vaccine constructs were developed based on the envelope glycoproteins of the virus (mainly gp120 and gp160), which are responsible for virus binding to the target cells, and serve as the main targets for the neutralizing antibodies. The first HIV vaccine trial conducted in the US started in 1988 and evaluated a recombinant form of gp160 produced in a baculovirus-insect cell system [4]. Other envelope constructs were designed, especially gp120 and gp160 molecules produced in mammalian cell systems. 

This period of the antibody paradigm was very active, with different lines of research being explored. These included the use of poxvirus vectors to prime the antibody responses [5]; the development of non-human primate (NHP) models for HIV vaccine research; the identification of different genetic subtypes of the virus [6]; the classification of R5 and X4 virus phenotypes [7]; and the finding that primary and cell-cultured isolates of HIV have different sensitivity to neutralizing antibodies *in vitro* [8]. Other avenues of research that were less productive included: the general agreement that the V3 loop of gp120 constituted the Principal Neutralization Domain (PND) of HIV [9]; the observation that NHP could be protected with whole inactivated SIV, which turned out be mediated by a xenoimmunization mechanism [10]; and the potential use of live attenuated vaccines [11].

Nevertheless, this period was characterized by the expectation that a vaccine would be developed within the next few years. That belief led to major efforts to prepare international sites for the conduct of vaccine efficacy trials [12]. This energy was also reflected by the creation in 1995 of the AIDS Vaccine Advocacy Coalition (AVAC), the challenge that President Clinton posed to the scientific community in 1997 to develop an HIV vaccine within 10 years [13]; and the establishment in 1996 of the International AIDS Vaccine Initiative (IAVI).

This first period came to an end in 2003, when the negative results of the VaxGen trials were reported. Those were the first two efficacy trials of any candidate vaccine, simultaneously conducted in Thailand (VAX003) and North America (VAX004), to test the protective efficacy of two different preparations of recombinant gp120 vaccines [14,15]. 

### 2.2. Second Wave of HIV Vaccine Trials: Induction of CTL Responses (1995–2007)

The failure of the VaxGen trials catalyzed a rethinking in the field with a re-examination of the scientific basis for HIV vaccine development. The second wave of HIV vaccine development began with the recognition in the early 2000s of the critical importance of CD8^+^ T-cell responses in the control of HIV infection [16]. This new paradigm led to the development and refinement of live recombinant viral vectors, especially poxvirus and adenovirus vectors, as well as of DNA vaccines.

On the organizational side, the VaxGen results stimulated the search of mechanisms for a more strategic and coordinated approach to solve the HIV vaccine challenge. This led to the eventual establishment of Global HIV Vaccine Enterprise, initially proposed in 2003 [17]. The Enterprise stimulated new investments in the field, including the launching in 2005 of the NIH-funded Center for HIV Vaccine Immunology (CHAVI) and of the Bill & Melinda Gates Foundation supported Collaboration for AIDS Vaccine Discovery (CAVD) in 2006. 

This period saw much work trying to understand the dynamics of cell mediated immunity (CMI) in natural infection and in animal models. Those studies provided strong evidence that cytotoxic T-lymphocytes (CTLs) were important in controlling virus replication in infected people, although not sufficient to completely eliminate the virus. Moreover, NHP protection experiments repeatedly showed that CTL-based SIV vaccines could not prevent acquisition of infection, although some of them decreased virus load and progression to disease in vaccinated animals that became infected after performing a virus challenge. This, combined with the conviction that gp120 vaccines would not offer significant protection against primary infection, led by 2007 to the conclusion that the best that could be done was to develop disease-modifying vaccines [18]. 

The field then turned to develop vaccines that stimulate CD8^+^ T cells in humans. Research done in the early 1990s has shown that recombinant plasmid DNA delivered into the skin or muscle induce viral specific immune responses. This relatively simple technology was seen as a potential modern replacement of live-attenuated vaccines, capable of inducing a whole range of immune responses. Starting in the mid-1990s, DNA technology began to be explored in the SIV/macaque model and in human trials. Very quickly, however, it was found that the robust immunogenicity observed in small animals did not translate to NHP or humans. To address the problem, different technologies were explored to enhance the immunogenicity of DNA vaccines including electroporation, co-administration with cytokines, and the use of prime-boost regimes [19].

During this period two viral vectors were preferentially used for the development of HIV vaccines, poxviruses and adenoviruses. Although poxviruses were the first to be used for HIV vaccine development [20], including the first poxvirus-prime/protein-boost trial conducted in the US in 1991 [21], the emphasis during this wave shifted to adenovirus vectors. A major driving force of the adenovirus vector effort was the pharmaceutical company Merck and Co, Inc. (Whitehouse Station, NJ, USA) which in 2001 announced results from their initial NHP protection experiments using a replication-defective adenovirus 5 (Ad5) vector expressing the SIV *gag* gene [22]. Based on those results, a candidate vaccine using a mixture of recombinant Ad5 vectors expressing the HIV *gag*, *pol* and *nef* genes moved in 2004 to two sequential efficacy trials: STEP, in the US and other countries in the Americas, and Phambili, in South Africa. Both trials were halted in September 2007 due to an interim review of the STEP trial that revealed the vaccine was not protective and that vaccination appeared to be associated with an increased risk of HIV acquisition in vaccinated individuals who had preexisting antibodies against Ad5 [23,24,25].

An early concern related to the potential use of Ad5 vectors was that the preexisting immunity to Ad5, which is quite prevalent especially in less developed countries, could impair its immunogenicity. This concern led to the development of alternative adenovirus vectors, either based on less prevalent human adenovirus serotypes or on simian adenoviruses for which no preexisting immunity exists in human populations [26]. 

It still remains unclear if the enhancement of HIV infectivity observed in the STEP trial is a common characteristic of all adenovirus vectors, or if it is a specific trait of the Ad5 vectors. Since different adenovirus serotypes have different biological properties, the answer to that question deserves additional research and consideration.

The negative results from the STEP trial came as a surprise to the scientific community who had high expectations for the cell-mediated immunity approach. In response, the scientific community reacted with a call to reconsider the clinical research strategy and to focus more on a basic research agenda [27].

### 2.3. Third Wave: Combinations of Different Immune Responses (from 2007)

The third wave of HIV vaccine development, aimed at exploring combinations of immune responses, was initiated after the disappointing results from the STEP trial were announced. However, two years after the STEP trial was stopped, surprisingly positive (although modest) results from the RV144 trial were reported, in October 2009. The trial was conducted among 16,402 adults in Thailand to test the protective efficacy of a prime-boost combination of two vaccines: a canarypox-HIV recombinant vector followed by a recombinant gp120 protein, and demonstrated a 31.2% efficacy in preventing HIV infection [28]. An unprecedented scientific collaboration was organized to try to identify potential immune correlates of protection, which generated the hypothesis that V1V2 antibodies may have contributed to protection, whereas high levels of Env-specific IgA antibodies may have mitigated the effects of the protective antibodies [29]. The analysis failed to identify neutralizing antibodies as a potential correlate, turning the attention to the potential role of non-neutralizing antibodies, probably those involved in antibody-dependent cell-mediate cytotoxicity (ADCC). Discussions are now underway to confirm and extend results from the RV144 trial to other populations in southern Africa and Thailand (through the so-called Pox-Protein Public Private Partnership, or P5) [30].

To some extent, the modest success obtained with the RV144 trial brought new attention to the importance of conducting clinical trials, especially efficacy trials, to complement the basic research effort. This, taken together with the failure of the CTL vaccine tested in the STEP trial, turned the HIV vaccine paradigm pendulum back to the induction of antibodies. 

The Holy Grail of HIV vaccine research has been the development of immunogens capable of eliciting broadly neutralizing antibodies (bnAb) that can protect against the large number of immunologically different strains of HIV that circulate globally [31]. It is known that roughly 20% of HIV infected individuals develop such bnAb, typically after two years of infection. As early as 1992, the first broadly neutralizing monoclonal antibodies (bnmAb) were isolated and characterized. In recent years there has been an explosion in the discovery of new bnmAb, targeting different epitopes in the HIV envelope glycoproteins. These epitopes are being explored as potential targets for vaccine development, an effort that has been facilitated by new knowledge on the molecular structure of the HIV envelope. However, a major challenge the field is confronting is the dissociation between antigenicity (the ability of a molecule to be recognized by given monoclonal antibodies) and immunogenicity (the ability of those molecules to induce in animals or humans the corresponding antibodies). In this regard, much has been learned in the last two years about the mechanisms for the development of broadly neutralizing antibodies [32]. The success of passive immunization experiments in animals, using different bnmAb, is stimulating research on the potential use of those antibodies for prevention and treatment of HIV infection in humans [33].

However, T-cell vaccines received a surprising boost in 2011, with the description of a profound early control of SIV by effector-memory T cells induced by a Rhesus cytomegalovirus (RhCMV) vectored vaccine [34,35]. 

On the other hand, the latest disappointment in the field was the stopping of the HVTN 505 trial in April 2013, for lack of efficacy [36]. The vaccine tested consisted of a prime-boost regimen involving DNA priming and boosting with Ad5 vectors.

## 3. Lessons Learned

Three decades of HIV vaccine research has taken the field through a roller coaster of many failures and a few modest successes [2]. It is important to take stock and to draw lessons from the past to avoid the definition of insanity attributed, among others, to Albert Einstein: “doing the same thing over and over again and expecting different results.”

In this regard, I recently discussed several lessons from the 1954–1955 field trial of the Salk inactivated polio vaccine, that could inform the development of an HIV vaccine, as follows [37]:

1. Paradigms change, and “expert” opinion can be wrong;
2. Basic science is essential, but it alone will not be sufficient to develop a vaccine;
3. Human data trump everything we do *in vitro* or in animal models;
4. Different vaccine concepts need to be tested in parallel;
5. The availability of other preventive interventions may decrease the interest on vaccine development;
6. Sustained support over the long term is needed;
7. Invest in the future by protecting the funding necessary for vaccine development; and
8. Preparation for success can shorten the time between vaccine development and public health impact.

This article specifically focuses on lessons learned after 30 years of HIV vaccine research. In order to have a more robust and informed discussion, I reached out to a number of colleagues. Thirty-six of whom responded with thoughtful comments (which are italicized in the text, unattributed) and their names are listed in the acknowledgment section. Nevertheless, the author assumes full responsibility for the views expressed. 

Perhaps, the first lesson is that “*we need to be willing to learn from the experiences of the past.*” The same advice was voiced by the Spanish-born poet and philosopher George Santayana (1863–1952), who reminded us that “Those who cannot remember the past are condemned to repeat it” [38]. 

### 3.1. The Development of an HIV Vaccine Is an Extremely Difficult Challenge

“*If it were a simple problem, someone would have solved it by now.*” The naivete of the first two decades of HIV vaccine research has now been replaced by the sobering conclusion that developing a HIV preventive vaccine is one of the most difficult challenges that biomedical research is confronting. “*The field* *has to move away from a home run philosophy*”, which seems to have equally affected researchers, funders and advocates. The priority over the last few years has been “*to win, not to think.*” A more systematic and coordinated approach to problem solving needs to be adopted. 

On the positive side, recent results from the RV144 trial suggest that developing a vaccine that prevents HIV acquisition is possible. However, there are more cautious voices that argue that in reality we do not know if we would be able to solve the problem, or when a practical vaccine will be developed. 

The reality is that after 30 years of HIV vaccine research “*we are mostly in the discovery phase*.” It is important to be guided by data and to “*resist the temptation of trusting our own beliefs, preconceived* *ideas and feelings of certainty.*” We constantly need to remind ourselves that “*good science (rational or empirical) matters*!” 

### 3.2. The Temptation of Just Following the Fashion Should Be Avoided

Preconceived ideas herded the first two waves of HIV vaccine development. As discussed above, the hepatitis B vaccine model guided the antibody paradigm wave of HIV vaccine development, which was followed by a second wave based on the conviction that protective responses observed in elite controllers could be directly translated into the development of a preventive vaccine. Although it made sense to explore those lines of research, the problem was that the entire field followed the fashion with an almost religious fervor. “*Everyone was running after the same ball, which sometimes changed direction.*” Funding agencies did the same, providing limited funding to explore alternative approaches. 

### 3.3. Clinical Trials Are Critical, Especially Large-Scale Efficacy Trials

“*HIV research has provided much knowledge but not a vaccine.*” Scientific knowledge needs to be translated into products for clinical trials.

Results from three efficacy trials (the two VaxGen trials reported in 2003 and the STEP trial in 2007) were determinant in changing the prevailing vaccine development paradigms. The current paradigm, which is exploring a broader range of immune responses, was reinforced by the 2009 results from the RV144 trial.

The current dilemma and a potential problem is that “*many scientists have built entire careers in HIV, which has become a research industry.*” In some cases there is little motivation to “*kill early and kill hard*,” with a reluctance to move fast into the clinic, where negative results could have detrimental consequences for grants or professional careers.

One critical lesson is that “*nothing replaces clinical trials*,” whose results are often “*unpredictable and surprising.*” Clinical trials are “*time-consuming, expensive and dependent on appropriate clinical trial infrastructure*.” In addition, the conduct of clinical trials, especially large scale trials “*can be very controversial*” and, often, “*decisions need to be made without first achieving consensus.*” However*,* “*judicious use of large scale efficacy trials*” is essential to advance the field. 

### 3.4. HIV Vaccine Research Requires Long-Term Commitment and Funding

The recent development of effective non-vaccine prevention intervention for HIV (such as microbicides and pre-exposure prophylaxis) is welcomed. These interventions would surely have a significant public health impact, at least in some populations, especially in developed countries. However, an HIV vaccine is believed to be needed to fully accomplish the goal of an AIDS-free world and we should “*avoid complacency because of the existence of those other HIV preventive interventions*.” 

A practical problem that the field needs to solve is how to conduct vaccine clinical trials in the context of other prevention interventions. These interventions would decrease HIV incidence in the trial population, making it more difficult to assess vaccine-induce protection.What we cannot afford is to give-up, this requiring “*long term perseverance and dedication of scientists and funders.*”

“*HIV vaccine development is not for the faint of heart.*” Younger generations of scientists are needed to continue the effort and “*new people need to be attracted to the field.*” However, there is a perception among some young scientists that the HIV field is “*too crowded for them to have any chance and it would be better to focus on a new research area*.” “*Unless they can see a career path we will fail to recruit the brightest and the best to this effort*.” I believe, however, that the HIV vaccine field presents to the young scientists the incredible challenge and opportunity to work on a major research challenge that for years has resisted solution, also contributing to the solution of one of the major global health problem of our time.

Given that “*classical vaccinology has not helped developing an HIV vaccine*,” the field has to keep an open mind, exploring innovative approaches that have not been explored before*.* In this regard, experience has shown that “*each new vaccine needs a champion.*”

### 3.5. Sustainable Collaborations Are Needed to Accelerate the Development of an HIV Vaccine

The development of an HIV vaccine is a complex scientific endeavor which requires multiple collaborations. Unfortunately, “*we have not applied all available knowledge because of the silo nature of science*,” although both “*big science and small science can and should complement each other*” [39]. Different modalities of collaborations have been established throughout the years, especially for the conduct of clinical trials where expanded access to trial populations is needed. Examples of these collaborations are the AIDS Vaccine Evaluation Groups (AVEG), the Preparation for AIDS Vaccine Evaluation (PAVE) initiative, and the HIV Vaccine Trials Network (HVTN). Most recently, the P5 partnership has brought together different players from the public, private and philanthropic sectors.

Other modalities of collaboration were also established to tackle upstream aspects of HIV vaccine research, including the IAVI’s Neutralizing Antibody Consortium, as well as the Center for HIV Vaccine Immunology (CHAVI) and the Collaboration for AIDS Vaccine Discovery (CAVD). 

Regional collaborations were also established, including the African AIDS Vaccine Programme/Partnership (AAVP) and the AIDS Vaccine for Asia Network. The 2003 proposal to establish the Global HIV Vaccine Enterprise as a mechanism to accelerate the development of an HIV vaccine was received with great enthusiasm and heralded a new era of intensified collaboration [17]. 

One of the most successful examples of true partnership has been the multiple collaborations established by different groups of Thai scientists and international collaborators, a commitment that spanned many years culminating with the RV144 trial [28,40,41]. 

However, an analysis of the “natural history” of many collaborative efforts reveals that after a period of initial excitement and support, the original goals of many of these efforts are forgotten or weakened and many of them fail to thrive. With time, some of these collaborative efforts lose strategic focus and/or financial support, remaining in place just as faint memories of unfulfilled hopes. As support for global health projects declines, the HIV vaccine field will have to be creative and commit to more coordination and collaboration [42].

The HIV vaccine field has been “*too inward looking*” and it is important to learn from other vaccine efforts. Future collaborations should learn from experience, ensuring “*clarity of objectives,*” “*industrial involvement and partnerships*,” “*full involvement of local investigators and communities*” and developing novel approaches to “*prioritize, synergize and interconnect.*”

## 4. Moving Forward

A major global effort to expand access to the existing prevention and therapeutic interventions has already resulted in a decrease in the number of new HIV infections, from 3.5 million in 2005 to 2.3 million in 2012. Ongoing work by UNAIDS indicates that an intensified effort to provide existing interventions to the appropriate target populations would result in additional reductions in HIV incidence, although it would not be able to bring the epidemic to zero. However, moving forward with that intensified effort would cost up to 23 billion US dollars per year, a financial commitment that would be challenging to maintain. Preliminary modeling work by IAVI, indicates that a 80% effective vaccine introduced in 2025 would be critical in significantly reducing the number of new HIV infections in an effort to achieve the goal of an AIDS free generation [43]. The world has invested almost 9.5 billion US dollars in HIV vaccine research since 2000, and the current annual level of investment dedicated to vaccine development is close to 850 million US dollars. That amount seems relatively modest, considering that a vaccine would be the most cost-effective intervention to control the HIV pandemic. However, additional work is needed to estimate the costs of rolling out a global HIV vaccination campaign.

Summarizing the many lessons from the past, I would like to make a set of personal recommendations. These recommendations are intended to stimulate the discussion and intellectual dialogue that the field needs to accelerate the development of an HIV vaccine.

1. Establish and maintain a program of truly innovative research with protected funding to explore out-of-the-paradigm approaches (perhaps not less than 10 percent of the total investment);
2. Continue the basic research effort, exploring novel opportunities to conduct translational research, including the implementation of small experimental medicine trials (small human trials designed to answer critical questions prior to embarking on formal product development activities);
3. Discuss an ambitious goal of initiating a certain number of well-coordinated efficacy trials in the next five years. Planning for these trials would help structure the discussion around scientific questions, vaccine manufacturing capacity, access to and preparation of trial populations, and funding issues;
4. Design appropriate strategies and trials to answer lingering questions in the field, such as the potential protective efficacy of vaccines against different HIV clades and routes of transmission, which differ in different geographic regions of the world;
5. Strengthen the global HIV vaccine architecture by supporting the role of different national, regional and global organizations (including WHO and UNAIDS) which have different audiences and constituencies. In particular, strengthen the role of the Global HIV Vaccine Enterprise as a venue where multiple partners plan their collaborative effort; and
6. Bring new partners to the HIV vaccine field and strengthen interactions with other organizations that work in the HIV prevention arena.

All of the above would need to be conducted with the necessary sense of urgency that the epidemic is imposing on us. 

## References

[1] Gallo R.C., Montagnier L. (1987). The chronology of AIDS research. Nature.

[2] Esparza J. (2013). A brief history of the global effort to develop a preventive HIV vaccine. Vaccine.

[3] Plotkin S.A. (2010). Correlates of protection induced by vaccination. Clin. Vaccine Immunol..

[4] Merz B. (1987). HIV vaccine approved for clinical trials. J. Amer. Med. Assoc..

[5] Hu S.L., Abrams K., Barber G.N., Moran P., Zarling J.M., Langlois A.J., Kuller L., Morton W.R., Benveniste R.E. (1992). Protection of macaques against SIV infection by subunit vaccines of SIV envelope glycoprotein gp160. Science.

[6] Myers G., Korber B., Wain-Hobson S., Kuan-Teh J., Henderson L.E., Pavlakis G.N. (1992). Human Retroviruses and AIDS.

[7] Berger E.A., Doms R.W., Fenyö E.M., Korber B.T., Littman D.R., Moore J.P., Sattentau Q.J., Schuitemacker H., Sodroski J., Weiss R.A. (1998). A new classification for HIV-1. Nature.

[8] Cohen J. (1994). The HIV vaccine paradox. Science.

[9] Bolognesi D.P. (1990). Progress in vaccine development against SIV and HIV. J. Acquir. Immune Defic. Syndr..

[10] Weiss R. (1991). Monkey business over AIDS vaccine. Br. Med. J..

[11] Daniel M.D., Kirchoff F., Czajak S.C., Segal P.K., Desrosiers R.C. (1992). Protective effects of a live-attenuated SIV vaccine with a deletion in the *nef* gene. Science.

[12] Esparza J., Osmanov S., Kallings L.O., Wigzell H. (1991). Planning for HIV vaccine trials: The World Health Organization perspective. AIDS.

[13] Lawler A., Cohen J. (1997). A deadline for an AIDS vaccine. Science.

[14] Flynn N.M., Forthal D.N., Harro C.D., Judson F.N., Mayer K.H., Para M.F. (2005). rgp 120 HIV Vaccine Study Group. Placebo-controlled phase 3 trial of a recombinant glycoprotein 120 vaccine to prevent HIV-1 infection. J. Infect. Dis..

[15] Pitisuttithum P., Gilbert P., Gurwith M., Heyward W., Martin M., van Griensven F., Hu D., Tappero J.W., Choopanya K. (2006). Randomized, double-blind, placebo-controlled efficacy trial of a bivalent recombinant glycoprotein 120 HIV-1 vaccine among injection drug users in Bangkok, Thailand. J. Infect. Dis..

[16] McMichael A., Hanke T. (2002). The quest for an AIDS vaccine: Is the CD8^+ ^T-cell approach feasible?. Nature Rev. Immunol..

[17] Klausner R.D., Fauci A.S., Corey L., Nabel G.J., Gayle H., Berkley S., Haynes B.F., Baltimore D., Collins C., Douglas R.G. (2003). The need for a global HIV vaccine enterprise. Science.

[18] Johnston M.I., Fauci A.S. (2007). An HIV vaccine—Evolving concepts. N. Eng. J. Med..

[19] Lu S. (2008). Immunogenicity of DNA vaccines in humans. Hum. Vaccines.

[20] Zagury D., Léonard R., Fouchard M., Réveil B., Bernard J., Ittelé D., Cattan A., Zirimwabagabo L., Kalumbu M., Justin W. (1987). Immunization against AIDS in humans. Nature.

[21] Graham B.S., Matthews T.J., Belshe R.B., Clements M.L., Dolin R., Wright P.F., Gorse G.J., Schwartz D.H., Keefer M.C., Bolognesi D.P. (1993). Augmentation of human immunodeficiency virus type 1 neutralizing antibody by priming with gp160 recombinant vaccinia and boosting with rgp160 in vaccinia-naive adults. The NIAID AIDS vaccine clinical trials network. J. Infect. Dis..

[22] Cohen J. (2001). Merck reemerges with a bold AIDS vaccine effort. Science.

[23] Cohen J. (2007). Promising AIDS vaccine’s failure leaves field reeling. Science.

[24] Buchbinder S.P., Mehrotra D.V., Duerr A., Fitzgerald D.W., Mogg R., Li D., Gilbert P.B., Lama J.R., Marmor M., del Rio C. (2008). Efficacy assessment of a cell-mediated immunity HIV-1 vaccine (the Step Study): A double-blind, randomised, placebo-controlled, test-of-concept trial. Lancet.

[25] Duerr A., Huang Y., Buchbinder S., Coombs R.W., Sanchez J., del Rio C., Casapia M., Santiago S., Gilbert P., Corey L. (2012). Extended follow-up confirms early vaccine-enhanced risk of HIV acquisition and demonstrates waning effect over time among participants in a randomized trial of recombinant adenovirus HIV vaccine (step study). J. Infect. Dis..

[26] Barouch D.H., Nabel G.J. (2005). Adenovirus vector-based vaccines for human immunodeficiency virus type 1. Hum. Gene Ther..

[27] Fauci A.S., Johnston M.I., Dieffenbach C.W., Burton D.R., Hammer S.M., Hoxie J.A., Martin M., Overbaugh J., Watkins D.I., Mahmoud A. (2008). HIV vaccine research: The way forward. Science.

[28] Rerks-Ngarm S., Pitisuttithum P., Nitayaphan S., Kaewkungwal J., Chiu J., Paris R., Premsri N., Namwat C., de Souza M., Adams E. (2009). Vaccination with ALVAC and AIDSVAX to prevent HIV-1 infection in Thailand. N. Engl. J. Med..

[29] Haynes B.F., Gilbert P.B., McElrath M.J., Zolla-Pazner S., Tomaras G.D., Alam S.M., Evans D.T., Montefiori D.C., Kamasuta C., Sutthent R. (2012). Immune-correlates analysis of an HIV-1 vaccine efficacy trial. N. Engl. J. Med..

[30] McEnery R. Understanding the P5 partnership. VAX Report.

[31] Montefiori D., Sattentau Q., Flores J., Esparza J., Mascola J. (2007). Working Group convened by the Global HIV Vaccine Enterprise. Antibody-based HIV-1 vaccines: Recent developments and future directions. PLoS Med..

[32] Haynes B.F., Kelsoe G., Harrison S.C., Kepler T.B. (2012). B-cell-lineage immunogen design in vaccine development with HIV-1 as a case study. Nat. Biotechnol..

[33] Klein F., Mouquet H., Dosenovic P., Scheid J.F., Scharf L., Nussenzweig M.C. (2013). Antibodies in HIV-1 vaccine development and therapy. Science.

[34] Hansen S.G., Ford J.C., Lewis M.S., Ventura A.B., Hughes C.M., Coyne-Johnson L., Whizin N., Oswald K., Shoemaker R., Swanson T. (2011). Profound early control of highly pathogenic SIV by an effector memory T-cell vaccine. Nature.

[35] Picker L.J., Hansen S.G., Lifson J.D. (2012). New paradigms for HIV/AIDS vaccine development. Annu. Rev. Med..

[36] National Institutes of Allergy and Infectious Disease Statement NIH Discontinues immunizations in HIV vaccine study. http://www.niaid.nih.gov/news/newsreleases/2013/Pages/HVTN505April2013.aspx.

[37] Esparza J. (2013). A tale of two vaccines: Lessons from polio that could inform the development of an HIV vaccine. AIDS.

[38] Santayana G. (1905). Life of Reason, Reason of Common Sense.

[39] Esparza J., Yamada T. (2007). The discovery value of big science. J. Exp. Med..

[40] Pitisuttithum P., Francis D., Esparza J., Thongocharoen P. (2006). HIV Vaccine Research and Development in Thailand.

[41] Pitisuttithum P., Choopanya K., Rerk-Ngnam S. (2010). HIV-vaccine research and development in Thailand: Evolution and challenges. Vaccine.

[42] Mairal A. Can collaboration resuscitate global health funding?. http://www.theguardian.com/global-development-professionals-network/2013/jul/31/publicprivate-partnerships-global-health-programmes.

[43] McGlynn M. Developing AIDS vaccines, policy perspectives. Proceeding of XIII AIDS Vaccine Conference.

